# Evaluation of myocardial strain in patients with myocardial amyloidosis using feature-tracking technique

**DOI:** 10.1186/1532-429X-18-S1-Q46

**Published:** 2016-01-27

**Authors:** Tarun Pandey, Alapati Sindhura, Mohan Mallikarjuna Rao Edupuganti, Shelly Lensing

**Affiliations:** 1 grid.241054.60000000122929177Radiology, University of Arkansas for Medical Sciences, Little Rock, AR USA; 2 grid.241054.60000000122929177Cardiology, University of Arkansas for Medical Sciences, Little Rock, AR USA; 3 grid.241054.60000000122929177Biostatistics, University of Arkansas for Medical Sciences, Little Rock, AR USA

## Background

Cardiac amyloidosis results in diffuse involvement of the myocardium. For non-invasive diagnosis of myocardial amyloidosis, delayed post contrast MRI is the current gold standard. However, myocardial involvement can be patchy; not infrequently the delayed enhancement images are technically limited and many patients cannot tolerate contrast due to poor renal function. Hence an alternative method for evaluation of myocardial amyloidosis is needed that would replace or supplement the current techniques. Myocardial strain analysis has been used in the past using echocardiography and grid tagged MRI. Echo is limited by spatial resolution and lack of 3D imagery and grid tagged strain analysis require prospective acquisition of grid tagged images. On the other hand, feature tracking strain analysis can utilize the routine multiplanar cine images for strain analysis, hence being quite easy to incorporate as part of routine cardiac protocol.

**Hypothesis:** Strain evaluation using feature tracking can be successfully utilized to diagnose amyloidosis. We hypothesize that global myocardial strain values should be reduced in patients with amyloidosis when compared to patients with normal cardiac function.

## Methods

A retrospective IRB approved review was performed of 63 patients who underwent cardiac MRI at our facility for evaluation of myocardial amyloidosis. Baseline, demographic and imaging parameters were recorded from chart review. The diagnosis of amyloidosis was established on tissue biopsy. Conventional short axis, vertical long axis and 4-chamber cine SSPF images from the cardiac MRI scans were used to generate 2D and 3D myocardial strain maps using myocardial feature tracking software. Global radial, circumferential and longitudinal stain values were computed.

## Results

Cardiac MR feature tracking strain analysis was able to differentiate patients with myocardial amyloidosis from those without myocardial amyloid with high sensitivity and specificity. There was significant reduction of global radial, circumferential and longitudinal stains in patients with myocardial amyloidosis using both 2D and 3D parameters (all p < 0.001). The area under the ROC curve for peak radial, circumferential and longitudinal strain were 89.5%, 91.3% and 88.1% using a cut off of 24.85, -15.12 and -11.59 respectively. The maximum sensitivity (89.3%) was achieved if any of the three parameters were abnormal, and the maximum specificity (85.7%) when all three parameters were abnormal.

## Conclusions

Global myocardial strain is significantly reduced in patients with cardiac amyloidosis. Cardiac MRI feature tracking global myocardial strain analysis is a robust tool for detection of cardiac amyloidosis.

